# Prevalence of malnutrition among HIV-infected children in Central and West-African HIV-care programmes supported by the *Growing Up Programme* in 2011: a cross-sectional study

**DOI:** 10.1186/s12879-015-0952-6

**Published:** 2015-05-26

**Authors:** Julie Jesson, David Masson, Arsène Adonon, Caroline Tran, Capitoline Habarugira, Réjane Zio, Léoncie Nicimpaye, Sophie Desmonde, Goreth Serurakuba, Rosine Kwayep, Edith Sare, Tiefing Konate, Abdoulaye Nimaga, Philemon Saina, Akossiwa Kpade, Andrée Bassuka, Gustave Gougouyor, Valériane Leroy, for the Growing Up Working Group

**Affiliations:** Inserm, Centre Inserm U897 - Epidémiologie - Biostatistiques, Bordeaux, France; Centre de Recherche Inserm U897, Institut de Santé Publique, d’Epidémiologie et de Développement (ISPED), Université Bordeaux Segalen Case 11, 146 rue Léo Saignat, 33076 Bordeaux, Cedex France; Growing Up Programme - Sidaction & Initiative Développement, Paris, France; RACINES (Recherches Actions Communautaires Initiatives pour un Nouvel Espoir), Cotonou, Bénin; ANSS (Association Nationale de Soutien aux Séropositifs et malades du Sida), Bujumbura, Burundi; APECOS (Association de Prise en Charge des Orphelins du Sida ), Bujumbura, Burundi; SWAA (Society for Women against AIDS in Africa ) - Burundi, Bujumbura, Burundi; SWAA Littoral (Society for Women against AIDS in Africa), Douala, Cameroon; CSAS (Centre Solidarité Action Sociale), Bouaké, Ivory Coast; ARCAD (Association de Recherche, de Communication et d’Accompagnement à Domicile des personnes vivant avec le VIH et le sida), Bamako, Mali; AKS (Association Kenedougou Solidarité ), Sikasso, Mali; ADN (Association Djenandoum Naasson), Moundou, Tchad; AMC (Aide Médicale et Charité), Lomé, Togo; EVT (Espoir Vie Togo), Lomé, Togo; CRIPS (Centre de Recherche de d’Information Pour la Santé ) - Togo, Lomé, Togo

**Keywords:** HIV, Children, Malnutrition, Nutritional support, Africa

## Abstract

**Background:**

The burden of malnutrition among HIV-infected children is not well described in sub-Saharan Africa, even though it is an important problem to take into account to guarantee appropriate healthcare for these children. We assessed the prevalence of malnutrition and its associated factors among HIV-infected children in HIV care programmes in Central and West-Africa.

**Methods:**

A cross-sectional study was conducted from September to December 2011 among the active files of HIV-infected children aged 2–19 years old, enrolled in HIV-care programmes supported by the Sidaction *Growing Up Programme* in Benin, Burundi, Cameroon, Côte d’Ivoire, Mali, Chad and Togo. Socio-demographics characteristics, anthropometric, clinical data, and nutritional support were collected. Anthropometric indicators, expressed in Z-scores, were used to define malnutrition: Height-for-age (HAZ), Weight-for-Height (WHZ) for children < 5 years and BMI-for-age (BAZ) for children ≥5 years. Three types of malnutrition were defined: acute malnutrition (WHZ/BAZ < -2 SD and HAZ ≥ -2 SD), chronic malnutrition (HAZ < -2 SD and WHZ/BAZ ≥ -2 SD) and mixed malnutrition (WHZ/BAZ < -2 SD and HAZ < -2 SD). A multinomial logistic regression model explored associated factors with each type of malnutrition.

**Results:**

Overall, 1350 HIV-infected children were included; their median age was 10 years (interquartile range [IQR]: 7–13 years), 49 % were girls. 80 % were on antiretroviral treatment (ART), for a median time of 36 months. The prevalence of malnutrition was 42 % (95 % confidence interval [95% CI]: 40-44 %) with acute, chronic and mixed malnutrition at 9 % (95% CI: 6–12 %), 26 % (95% CI: 23–28 %), and 7 % (95% CI: 5–10 %), respectively. Among those malnourished, more than half of children didn’t receive any nutritional support at the time of the survey. Acute malnutrition was associated with male gender, severe immunodeficiency, and the absence of ART; chronic malnutrition with male gender and age (<5 years); and mixed malnutrition with male gender, age (<5 years), severe immunodeficiency and recent ART initiation (<6 months). Orphanhood and Cotrimoxazole prophylaxis were not associated with any type of malnutrition.

**Conclusions:**

The prevalence of malnutrition in HIV-infected children even on ART remains high in HIV care programmes. Anthropometric measurements and appropriate nutritional care of malnourished HIV-infected children remain insufficient and a priority to improve health care of HIV-infected children in Africa.

## Background

In 2012, 3.3 million children were living with Human Immunodeficiency Virus (HIV) worldwide, with more than 90 % in sub-Saharan Africa [1]. Regardless of HIV, sub-Saharan Africa is also the region of the world the most seriously affected by malnutrition, 21 % of children under 5 years are underweight, 39 % are stunted, and 9 % are wasted [2]. Malnutrition is the underlying cause of death among 35 % of children aged <5 years [3], and could lead to irreversible damages such as cognitive impairment, chronic diseases and growth failure [4].

Therefore, malnutrition is a major problem for children and especially for HIV-infected children since it creates a vicious circle with HIV infection. Indeed, on the one hand, malnutrition worsens HIV disease as it has similar effects on the immune system as HIV infection. For example, among malnourished people, lymphoid tissues are damaged, and CD4 T-cell concentration **is** decreased [5]. Deficiencies in vitamins and minerals contribute to oxidative stress, which can accelerate immune cell death [6] and increase HIV replication [7]. On the other hand, HIV infection increases the risk of malnutrition, because of a high pro-inflammatory cytokine activity which can cause growth impairment among children [8]. HIV-related opportunistic infections such as persistent diarrhoea or oral and oesophageal candidiasis have a negative impact on nutritional status among children [9]. HIV infection can also indirectly affect the child’s nutritional status, when it has an impact on the child’s social environment. In some contexts, when HIV concerns the most productive members of the family, the household economic capacities and the agricultural production are reduced, leading to a situation of food insecurity [10]. Furthermore, poor weaning practices among HIV-infected mothers can also have an impact on the child’s nutritional status [11].

Thus, malnutrition is a common complication among HIV-infected children. Low weight-for-age has been reported in up to 50 % of untreated HIV-infected children in resource-limited settings [12]. Among children with severe malnutrition, mortality risk is three times higher in HIV-infected children than in non-HIV-infected children [13]. Thus, nutritional care is fully part of the paediatric HIV healthcare package. The World Health Organisation recommends that an asymptomatic HIV-infected child should increase his energy requirements by 10 %, compared to a non-infected child; this is extended to 20 to 30 % during symptomatic HIV infection or episodes of opportunistic infections, and up to 50 to 100 % when a severe malnutrition episode occurs [14]. However, the burden of malnutrition remains difficult to quantify in HIV-infected people, most of all in children. A better understanding of this problem and its associated factors is necessary to improve HIV paediatric healthcare, especially in sub-Saharan Africa. Thus, we conducted a cross-sectional study, to assess the prevalence and associated factors of acute and chronic malnutrition among HIV-infected children followed up in the HIV-care programmes in Central and West Africa funded by the Growing Up programme.

## Methods

### Study population

The Growing Up programme is supported by two French NGOs: Sidaction and Initiative Développement, and supports 17 associations in 10 Central and West-African countries, taking care of HIV-infected children and their families through a comprehensive approach. Twelve of the associations participated in the study, in seven African countries: Benin, Burundi, Cameroon, Côte d’Ivoire, Mali, Chad and Togo. Nine of these associations are located in capital cities and three in other major cities, mainly in urban or peri-urban areas. After HIV diagnosis, children received medical treatment (Cotrimoxazole prophylaxis, treatment of acute opportunistic infections, antiretroviral therapy [ART] if eligible according to the 2010 WHO guidelines [15], and nutritional support). Children were followed-up at least every 2 months.

Nutritional support was usually provided to severely malnourished children identified according to the sites modalities, usually composed of, either an enriched flour or flour-sugar-oil mixture, or ready-to-use therapeutic foods such as Plumpy nut. For every centre, nutritional support was mainly used for children under two years of age to assist the weaning period. No specific nutritional protocol was defined for older children. The nutritional assessment was not yet routinely implemented at the time of the survey.

Data from each centre were collected and entered into a database with the formal approval of each participating clinical site. There were neither extra exams nor blood draws, nor extra data collection compared to the standard of care offered in each site. This study has been conducted in accordance with the principles of the Declaration of Helsinki of the World Medical Association. Parent’s verbal consent was collected during the conduct of the study, and all data records analysed in the database were anonymized.

### Study design

A cross-sectional study was conducted between September and December 2011 among all HIV-infected children enrolled in 12 of the *Growing Up Programme* partnering associations. Children included in the study were those with a confirmed HIV-infection (a positive serology for children older than 18 months, or a positive polymerase chain reaction [PCR] whatever the age), aged between 2 and 19 years old, ART-treated or not, with available data for gender, age, weight and height and HIV care at the time of the survey, and who had been seen at least once in the programme during the study period.

### Data management and variables

Data collection was standardised for each participating site, with a fact sheet. Data were collected during the follow-up routine visits and extracted from the medical records to be further centralised in a global database. Several types of data were collected: weight and height, measured during the survey visit according to the WHO recommendations [14], age during the survey expressed in categorical form ([2–5[, [5–10[, and [10–19[ years), the last CD4 count in cells/μL or in % less than 6 months before the study, clinical stage defined by the 2006 WHO guidelines [16], orphan status, information on HIV treatment, type of ART regimen and its duration (more or less than 6 months) and cotrimoxazole prophylaxis, and the type of the nutritional support (flour, powdered milk, solid or semi-solid foods, or Ready-to-Use Therapeutic Food [RUTF]) received during the study period and during the last six months prior to the study period. If a child had received at least one nutritional support before the study, we hypothesised that he had suffered from malnutrition and created a variable “malnutrition history”. CD4 was used in percentage for children < 5 years and in cells/μL for children ≥ 5 years and we defined immunodeficiency for age according to the 2006 WHO definitions [16]. Severe immunodeficiency was defined by CD4% < 15 or CD4/μL < 350, and moderate immunodeficiency by CD4% = [15–25[, or CD4/μL = [350–499[.

To define malnutrition, several anthropometric indicators are used according to WHO definitions: Height-for-Age, for children up to 19 years, Weight-for-Height for children < 5 years and BMI-for-Age for children ≥ 5 years, and Weight-for-age, for children < 10 years. These indicators are standardised using Z-scores, which quantify how many Standard Deviations (SDs) child’s weight and height is from the median value of a child of the same age and sex, in a reference population. For this analysis, we used the 2006 WHO growth charts for children <5 [17], and the 2007 WHO growth charts for children ≥ 5 [18]. Each indicator allows to define three types of malnutrition: wasting when Weight-for-Height Z-score (WHZ) or BMI-for-Age Z-score (BAZ) < -2 SD, stunting when Height-for-Age Z-score (HAZ) < -2 SD, and underweight when Weight-for-Age Z-score (WAZ) < -2 SD. A child is defined as moderately malnourished if the Z-score is between -3 and -2 SD, and severely malnourished if the Z-score < -3 SD. Z-scores were calculated using WHO Anthro Software (version 3.2.2, January 2011) and WHO AnthroPlus.

In this study, we combined these indicators to define three categories of malnutrition: (1) acute malnutrition defined by WHZ or BAZ < -2 SD and HAZ ≥ -2 SD; (2) chronic malnutrition defined by WHZ/BAZ ≥ -2 SD and HAZ < -2 SD, and (3) mixed malnutrition as WHZ/BAZ < -2 SD and HAZ < -2 SD. WAZ was not used here.

### Statistical analysis

Characteristics of the HIV-infected children included were first described by age group, then by the type of malnutrition (acute, chronic, and mixed malnutrition).

Comparisons were made using the Pearson χ2 test for qualitative variables and the Kruskal-Wallis test for quantitative variables. Prevalences of malnutrition according to the three anthropometric indicators were calculated with their 95 % confidence interval. A multinomial regression model was fitted to study the associated factors to the three types of malnutrition. All explanatory variables with P < 0.25 in bivariate analyses were selected for multivariate analyses. Potential explanatory variables included age group, gender, immunodeficiency for age, history and duration on ART, malnutrition history, orphan status, cotrimoxazole prophylaxis and country. Missing data were conserved in the analysis, creating a separate modality. Because WHO clinical stage is determined by severe clinical manifestations including malnutrition status [16], we chose to exclude this variable from multivariate analyses.

## Results

### Characteristics of the population

**Fig. 1:**
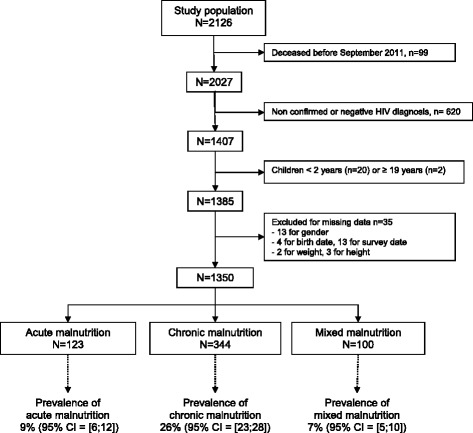
Selection of the population and prevalence of acute, chronic and mixed malnutrition (95 % Confidence Interval [CI])

Between September and December 2011, 2027 children were seen in the 12 participating centres of the *Growing up Programme*, representing more than 90 % of the active files. Among them, 1407 (69 %) had a confirmed diagnosis of HIV-infection. Of these children, 22 % were excluded for age criteria and 35 % for missing data. Finally, 1350 HIV-infected children were included in our study (Fig. 1). Their median age was 10 years (interquartile range [IQR] = [7–13]), 49 % were girls, 60 % were orphans for one or both parents, 77 % were on cotrimoxazole prophylaxis and 80 % were on ART for a median duration of 36 months (IQR = [18–61]). Of these children, 22 % had reached WHO clinical stage III or IV of HIV disease and 17 % were severely immunodeficient. Among the 237 children not on ART at the time of the study, 13 % were eligible (stage 3 or 4, or severely immunodeficient). More than 55 % of the included children did not receive any nutritional support at the time of the study or in the past 6 months (Table 1). Among the 45 % of children receiving nutritional support at inclusion, less than 2 % had received RUTF; and solid or semi-solid foods were the most frequently used (>80 %).

**Table 1:** Characteristics of the 1350 HIV-infected children of the study population according to age groups

Variables	Age group in years						*P*-value*	Total (*n* = 1350)	
	[2–5]		[5–10]		[10–19]				
	(*n* = 161)		(*n* = 505)		(*n* = 684)				
Gender, n, %							0.839		
Female	78	48	254	50	333	49		665	49
Male	83	52	251	50	351	51		685	51
CD4%, median, IQR^†^	31	24–37	-	-	-	-	-	31	24–37
CD4/mm3, median, IQR^‡^	-	-	788	505–1162	556	362–797	<0.001	642	412–952
Immunodeficiency by age^§^ , n, %							<0.001		
No immunodeficient	57	35	363	72	382	56		802	59
Moderate	15	9	52	10	129	19		196	15
Severe	6	4	65	13	152	22		223	17
Missing	83	52	25	5	21	3		129	10
WHO Clinical stage^§^, n, %							0.002		
I	70	44	223	44	380	56		673	50
II	48	30	137	27	157	23		342	25
III	25	16	89	18	98	14		212	16
IV	16	10	40	8	34	5		90	7
Missing	2	1	16	3	15	2		33	2
Nutritional support within 6 months prior to the study^||^, n, %							<0.001		
None	59	37	246	49	399	58		704	52
1 support	54	34	152	30	144	21		350	26
2 supports	15	9	34	7	51	8		100	7
3–4 supports	31	19	65	13	82	12		178	13
Missing	2	1	8	2	8	1		18	1
Nutritional support during the study^||^, n, %							<0.001		
None	68	42	261	52	418	61		747	55
1 support	55	34	161	32	157	23		373	28
2 supports	18	11	53	11	72	11		143	11
3 supports	17	11	25	5	31	5		73	5
Missing	3	2	5	1	6	1		14	1
Treatment, n, %							<0.001		
No treatment	4	3	13	3	14	2		31	2
Cotrimoxazole only	40	25	107	21	73	11		220	16
ART only	25	16	107	21	131	19		263	20
Cotrimoxazole + ART	90	56	271	54	459	67		820	61
Missing	2	1	7	1	7	1		16	1
Orphans status, n, %							<0.001		
Single (father deceased)	32	20	110	22	136	20		278	21
Single (mother deceased)	12	8	71	14	130	19		213	16
Double	2	1	62	12	248	36		312	23
No orphan	115	71	255	51	158	23		528	39
Missing	0	0	7	1	12	2		19	1
Country, n, %							<0.001		
Benin	5	3	25	5	18	3		48	4
Burundi	15	9	108	21	333	49		456	34
Cameroun	7	4	24	5	25	4		56	4
Côte d’Ivoire	43	27	100	20	104	15		247	18
Mali	40	25	79	16	54	8		173	13
Tchad	27	17	50	10	43	6		120	9
Togo	24	15	119	24	107	16		250	19

*Chi-square test for qualitative variables, Kruskal-Wallis test for quantitative variables

^†^children < 5 years, n’ = 78, ^‡^children ≥5 years, n’ = 480 for 5–10 years, n’ = 663 for 10–19 years, ^§^WHO 2006 guidelines, ^||^Number of different nutritional support given (flour, powdered milk, solid or semi-solid foods, RUTF)

Except for gender, all children characteristics differed significantly according to age groups (Table 1). More than half of the children between 2 and 5 years had missing CD4 data. The 2–5 and 5–10 years groups were at a more advanced clinical stage of HIV disease than the 10–19 years group (25 % vs 19 % at clinical stage III or IV, p = 0.002). Compared with the 5–10 and 10–19 years group**s**, the 2–5 years group had received more important nutritional support prior to the study (19 % vs 12–13 % with at least 3 supports, p < 0.001) and also during the study (11 % vs 5 % with 3 supports, p < 0.001). The youngest children were also less often orphans compared with older children (71 % of 2–5 years no orphans vs 51 % of 5–10 years vs 23 % of 10–19 years, p < 0.001) (Table 1).

### Prevalence of malnutrition

In the overall study population, 42 % of children were malnourished with 123 children (9 %, 95% CI = [6–12]) suffering from acute malnutrition, 344 (26 %, 95% CI = [23–28]) from chronic malnutrition, and 100 (7 %, 95% CI = [5–10]) from mixed malnutrition (Fig. 1). In other words, 16 % of children were wasted and 33 % were stunted with, in both cases, 36 % of them severely malnourished.

The prevalence of malnutrition differed significantly by age. Among children aged 2–5 years, half were malnourished, and we observed the highest rate of chronic malnutrition among this age group reaching 37 % (compared to 24 % in both 5–10 and 10–19 year old groups). Children aged 5–10 years were malnourished in 36 % of cases, and children aged 10 to 19 years in 44 % (Table 2).

**Table 2:** Prevalence of malnutrition among the 1350 HIV-infected children of the study population according to age groups

Malnutrition degree^†^, n, %, (IC95%)	Age group in years (*p*-value <0.001*)											
	[2–5]			[5–10]			[10–19]			Total		
	(*n* = 161)			(*n* = 505)			(*n* = 684)			(*n* = 1350)		
Acute malnutrition	10	6	(0;14)	44	9	(4;13)	69	10	(6;14)	123	9	(6;12)
Chronic malnutrition	59	37	(30;43)	122	24	(20;28)	163	24	(20;27)	344	26	(23;28)
Mixed malnutrition	13	8	(0;13)	17	3	(0;8)	70	10	(7;14)	100	7	(5;10)

*Chi-square test for the comparison of malnutrition prevalence according to age groups

^†^acute malnutrition : Weight-for-Age Z-score (WHZ)/BMI-for-age Z-score (BAZ) < -2 SD and Height-for-Age Z-score (HAZ) ≥ -2 SD, chronic malnutrition: WHZ/BAZ ≥ -2 SD and HAZ < -2 SD, mixed malnutrition: WHZ/BAZ and HAZ < -2 SD

Among the non-malnourished children, 45 % received at least one nutritional support before or during the study. Among the malnourished children at the time of the survey, whatever the type of malnutrition, 53 % received at least one nutritional support before or during the study. This nutritional support was more frequent for children between 2 and 5 years of age with no malnutrition, acute or chronic malnutrition, compared with older children (p < 0.001, p = 0.001 and p = 0.005 respectively). Children with mixed malnutrition and aged between 5 and 10 had more frequently a nutritional support compared with the other age groups (p = 0.025). Also, among children who had a nutritional support, most of them were supported both before and during the study, whatever the malnutrition degree (Table 3).

**Table 3:** Nutritional supplementation practices according to the type of malnutrition and age groups. *N* = 1350

Children on nutritional support according to malnutrition degree*, n, %,	Age group in years						Total		*P*-value^†^
	[2–5]		[5–10]		[10–19]				
	(*n* = 161)		(*n* = 505)		(*n* = 684)		(*n* = 1350)		
No malnutrition	*N* = 79		*N* = 322		*N* = 382		*N* = 783		
Nutritional support before the study only^‡^	9	11	14	4	10	3	33	4	<0.001
Nutritional support during the study only	2	3	10	3	6	2	18	2	
Nutritional support before and during the study	42	53	137	43	123	32	302	39	
Total nutritional support	53	67	161	50	139	36	353	45	<0.001
Acute malnutrition	*N* = 10		*N* = 44		*N* = 69		*N* = 123		
Nutritional support before the study only	2	20	3	7	4	6	9	7	0,123
Nutritional support during the study only	0	0	1	2	1	1	2	2	
Nutritional support before and during the study	6	60	16	36	20	29	42	34	
Total nutritional support	8	80	20	45	25	36	53	43	0.001
Chronic malnutrition	*N* = 59		*N* = 122		*N* = 163		*N* = 344		
Nutritional support before the study only	3	5	6	5	7	4	16	5	0.667
Nutritional support during the study only	1	2	1	1	0	0	2	1	
Nutritional support before and during the study	33	56	64	52	81	50	178	52	
Total nutritional support	37	63	71	58	88	54	196	57	0.005
Mixed malnutrition	*N* = 13		*N* = 17		*N* = 70		*N* = 100		
Nutritional support before the study only	1	8	1	6	5	7	7	7	0.310
Nutritional support during the study only	2	15	0	0	2	3	4	4	
Nutritional support before and during the study	4	31	10	59	27	39	41	41	
Total nutritional support	7	54	11	65	34	49	52	52	0.025

*acute malnutrition : Weight-for-Age Z-score (WHZ)/BMI-for-age Z-score (BAZ) < -2 SD and Height-for-Age Z-score (HAZ) ≥ -2 SD, chronic malnutrition: WHZ/BAZ ≥ -2 SD and HAZ < -2 SD, mixed malnutrition: WHZ/BAZ and HAZ < -2 SD

^†^Comparison of the nutritional support according to age groups and for each type of malnutrition, distinguishing time to support or not, **Chi**-square tests and Fisher tests if conditions of application not respected

^‡^In the last 6 months before the study

### Factors associated with acute malnutrition

More than half of children suffering from acute malnutrition were aged > 10 years; 63 % were boys, 37 % were known to be either moderately or severely immunodeficient and 27 % were reported to be at a WHO clinical stage III or IV, although unexplained moderate or severe malnutrition are criteria for classifying an HIV-infected child at these stages. Furthermore, 63 % of children presenting acute malnutrition didn’t receive any nutritional support during the study. Moreover, 66 % were initiated on ART for more than 6 months and 20 % were not yet receiving ART (Table 4).

**Table 4:** Baseline characteristics of the study population according to the type of malnutrition. *N* = 1350

Variables	Acute malnutrition*		Chronic malnutrition		Mixed malnutrition		Study population	
	(*N* = 123)		(*N* = 344)		(*N* = 100)		(*N* = 1350)	
	N	%	N	%	N	%	N	%
Age group in years								
[2–5]	10	8	59	17	13	13	161	12
[5–10]	44	36	122	35	17	17	505	37
[10–19]	69	56	163	47	70	70	684	51
Gender								
Female	45	37	155	45	33	33	665	49
Male	78	63	189	55	67	67	685	51
Immunodeficiency for age^‡^								
No immunodeficient	70	57	195	57	45	45	802	59
Moderate	13	11	46	13	14	14	196	15
Severe	33	27	57	17	31	31	223	17
Missing	7	6	46	13	10	10	129	10
WHO clinical stage^‡^								
I	50	41	139	40	43	43	673	50
II	34	28	101	29	23	23	342	25
III	16	13	73	21	24	24	212	16
IV	17	14	24	7	8	8	90	7
Missing	6	5	7	2	2	2	33	2
Nutritional support within 6 months prior to the study^||^								
None	69	56	148	43	51	51	704	52
1 support	21	17	100	29	19	19	350	26
2 supports	12	10	29	8	9	9	100	7
3–4 supports	18	15	65	19	20	20	178	13
Missing	3	2	2	1	1	1	18	1
Nutritional support during the study^||^								
None	77	63	161	47	54	54	747	55
1 support	21	17	114	33	22	22	373	28
2 supports	17	14	36	11	12	12	143	11
3 supports	6	5	30	9	11	11	73	5
Missing	2	2	3	1	1	1	14	1
Duration of ART								
<6 Months	11	9	30	9	12	12	94	7
≥6 Months	81	66	245	71	71	71	979	73
No yet started	25	20	63	18	12	12	237	18
Unknown	6	5	6	2	5	5	40	3
Cotrimoxazole prophylaxis								
Yes	101	82	271	79	74	74	1040	77
No	21	17	69	20	26	26	294	22
Unknown	1	1	4	1	0	0	16	1
Orphan status								
Single (father deceased)	17	14	67	20	23	23	278	21
Single (mother deceased)	21	17	62	18	19	19	213	16
Double orphan	33	27	70	20	26	26	312	23
No orphan	51	42	141	41	29	29	528	39
Missing	1	1	4	1	3	3	19	1
Country								
Benin	5	4	17	5	3	3	48	4
Burundi	34	28	104	30	30	30	456	34
Cameroun	0	0	16	5	0	0	56	4
Côte d’Ivoire	26	21	60	17	29	29	247	18
Mali	24	20	47	14	19	19	173	13
Chad	7	6	41	12	4	4	120	9
Togo	27	22	59	17	15	15	250	19

*acute malnutrition : Weight-for-Age Z-score (WHZ)/BMI-for-age Z-score (BAZ) < -2 SD and Height-for-Age Z-score (HAZ) ≥ -2 SD, chronic malnutrition: WHZ/BAZ ≥ -2 SD and HAZ < -2 SD, mixed malnutrition: WHZ/BAZ and HAZ < -2 SD

^†^ **Chi**-square test, ^‡^WHO 2006 guidelines, ^||^Number of different nutritional supports given (flour, powdered milk, solid or semi-solid foods, RUTF)

In univariate analysis, acute malnutrition was significantly twice as high in boys as in girls, and in children with severe immunodeficiency compared to those not (OR = 2.13, 95% CI = [1.44–3.16] and OR = 2.27, 95% CI = [1.43–3.62] respectively) (Table 5).

**Table 5:** Factors associated with malnutrition (acute, chronic and mixed), univariate and multivariate multinomial logistic regressions. *N* = 1350

Variable	Acute malnutrition*				Chronic malnutrition				Mixed malnutrition			
	(*N* = 123)				(*N* = 344)				(*N* = 100)			
	OR^†^	95% CI	aOR^†^	95% CI	OR^†^	95% CI	aOR	95% CI	OR^†^	95% CI	aOR	95% CI
Age group in years												
[2–5]	1	-	1	-	1	-	1	-	1	-	1	-
[5–10]	1.08	(0.52–2.24)	0.91	(0.39–2.15)	0.51	(0.34–0.75)	0.61	(0.38–0.99)	0.32	(0.15–0.69)	0.34	(0.14–0.84)
[10–19]	1.43	(0.70–2.89)	1.57	(0.66–3.78)	0.57	(0.39–0.84)	0.78	(0.47–1.29)	1.11	(0.59–2.11)	1.32	(0.56–3.09)
Gender (Male/Female)	2.13	(1.44–3.16)	2.27	(1.52–3.41)	1.50	(1.16–1.94)	1.56	(1.20–2.03)	2.50	(1.61–3.88)	2.60	(1.64–4.10)
Immunodeficiency by age^‡^												
No immunodeficient	1	-	1	-	1	-	1	-	1	-	1	-
Moderate	0.75	(0.40–1.39)	1.19	(0.47–3.02)	0.94	(0.65–1.38)	0.89	(0.60–1.31)	1.24	(0.66–2.34)	0.99	(0.51–1.91)
Severe	227	(1.43–3.62)	2.07	(1.25–3.42)	1.41	(0.98–2.03)	1.40	(0.96–2.06)	3.32	(2.01–5.51)	2.43	(1.40–4.23)
Missing	0.75	(0.33–1.69)	0.61	(0.23–1.63)	1.76	(1.17–2.65)	1.32	(0.78–2.23)	1.66	(0.80–3.44)	1.19	(0.47–3.02)
Duration of ART												
≥6 months	1	-	1	-	1	-	1	-	1	-	1	-
6 months	1.93	(0.95–3.90)	1.80	(0.84–3.89)	1.74	(1.06–2.85)	1.58	(0.93–2.67)	2.40	(1.21–4.78)	2.54	(1.17–5.55)
No yet started	1.31	(0.81–2.13)	1.70	(1.01–2.84)	1.09	(0.78–1.53)	1.11	(0.78–1.58)	0.72	(0.38–1.36)	1.11	(0.56–2.18)
Missing	1.87	(0.74–4.74)	2.48	(0.92–6.63)	0.62	(0.25–1.54)	0.69	(0.27–1.77)	1.78	(0.66–4.83)	2.34	(0.79–6.91)
Malnutrition history (Yes/No)^§^	0.95	(0.64–1.39)	1.23	(0.75–2.04)	1.73	(1.34–2.23)	1.99	(1.43–2.77)	1.23	(0.81–1.87)	1.67	(0.96–2.89)
Orphan status (Yes/No) ||	0.91	(0.62–1.34)	0.84	(0.55–1.29)	0.93	(0.72–1.20)	1.03	(0.77–1.37)	1.58	(1.00–2.49)	1.39	(0.83–2.32)
Treatment Cotrimoxazole (/No)												
Yes	1.44	(0.88–2.37)	-	-	1.18	(0.86–1.61)	-	-	0.85	(0.53–1.37)	-	-
Missing	0.77	(0.10–6.27)	-	-	0.94	(0.29–3.05)	-	-	<0.001	-	-	-

*acute malnutrition : Weight-for-Age Z-score (WHZ)/BMI-for-age Z-score (BAZ) < -2 SD and Height-for-Age Z-score (HAZ) ≥ -2 SD, chronic malnutrition: WHZ/BAZ ≥ -2 SD and HAZ < -2 SD, mixed malnutrition: WHZ/BAZ and HAZ < -2 SD

^†^OR = Odds Ratio, aOR = adjusted Odds Ratio, analyses adjusted on clinical centres

^‡^WHO 2006 guidelines

^§^Nutritional support 6 months prior the study

^||^Orphan status including both double and single orphans

In the adjusted analysis for age group, sex, country, immunodeficiency, malnutrition history, duration on ART and orphan status, boys were twice more likely malnourished than girls (aOR = 2.27, 95% CI = [1.52–3.41]), as well severely immunodeficient children compared to non-immunodeficient children (aOR = 2.07, 95% CI = [1.25–3.42]), and non-ART-treated children compared with those on ART for more than 6 months (aOR = 1.70, 95% CI = [1.01–2.84]) (Table 5).

### Factors associated with chronic malnutrition

Among children suffering from chronic malnutrition, 47 % were aged >10 years, 55 % were boys, 30 % were moderately or severely immunodeficient and 28 % were reported to be at and advanced clinical stage (III or IV). Among these children, 43 % hadn’t received any nutritional support during the 6 months prior to the study, 9 % were recently initiated on ART and 18 % were not receiving ART (Table 4).

In univariate analysis, chronic malnutrition was significantly twice as low in children older than 5 years of age as in younger children ([5–10[ vs. [2–5[ : OR = 0.51, 95% CI = [0.34–0.75], [10–19[ vs. [2–5[: OR = 0.57, 95% CI = [0.39–0.84]), higher in boys compared to girls (OR = 1.50, 95% CI = [1.16–1.94]), in children with missing immunological data (OR = 1.76, 95% CI = [1.17–2.65]), in children ART-initiated for less than 6 months compared to children on ART since more than 6 months (OR = 1.74, 95% CI = [1.06–2.85]), and in those who had an history of malnutrition (OR = 1.73, 95% CI = [1.34–2.23]) (Table 5).

In the adjusted analysis for age group, sex, country, immunodeficiency, malnutrition history, duration on ART and orphan status, the risk of chronic malnutrition was reduced in children aged 5–10 years compared to those aged 2–5 years (aOR = 0.61, 95% CI = [0.38–0.99]). On the other hand, chronic malnutrition was more likely among boys compared to girls (aOR = 1.56, 95% CI = [1.20–2.03]). Children who had received nutritional support within the 6 months prior to the study were more likely malnourished compared to those not receiving any support (aOR = 1.99, 95% CI = [1.43–2.77]) (Table 5).

### Factors associated with mixed malnutrition

Among children suffering from mixed malnutrition, 87 % were aged more than 10 years. There were 67 % of boys, 45 % were moderately or severely immunodeficient and 32 % were at an advanced clinical stage. Furthermore, 54 % hadn’t received any nutritional support during the study and 51 % hadn’t received any during the 6 months prior to the study. Moreover, 12 % were recently initiated on ART and 12 % were not receiving ART (Table 4).

In univariate analysis, mixed malnutrition was significantly twice as low in children aged 5 to 10 years of age as in younger children (OR = 0.32, 95% CI = [0.15–0.69]), higher in boys compared to girls (OR = 2.50, 95% CI = [1.61–3.88]), as well as in children with severe immunodeficiency (OR = 3.22, 95% CI = [2.01–5.51]), and in children ART-initiated for less than 6 months compared to children on ART for more than 6 months (OR = 2.40, 95% CI = [1.21–4.78]) (Table 5).

In the adjusted analysis for age group, sex, country, immunodeficiency, malnutrition history, duration on ART and orphan status, we observed lower risks of mixed malnutrition in children aged 5–10 years compared to 2–5 years (aOR = 0.34, 95% CI = [0.14–0.84]). Risks of mixed malnutrition were higher among boys compared to girls (aOR = 2.60, 95% CI = [1.64–4.10]) and among severely immunodeficient children compared to non immunodeficient children (aOR = 2.43, 95% CI = [1.40–4.23]) and in those on ART for less than 6 months compared to children on ART for more than 6 months (aOR = 2.54, 95% CI = [1.17–5.55]) (Table 5).

## Discussion

In this cross-sectional study conducted in 2011 among 1350 HIV-infected children receiving paediatric care in 12 associations of the *Growing up Programme* in sub-Saharan Africa, we documented a high prevalence of 42 % of malnutrition, with acute, chronic and mixed malnutrition which were 9 %, 26 % and 7 % respectively. This study provides also a snapshot of the nutritional practices in field conditions. Among the malnourished children in this study, more than half of the children didn’t receive any nutritional support during the study or within the 6 months prior to the study. Regarding associated factors, we report higher prevalence of malnutrition, whatever the type, among boys compared to girls. Children between 2 and 5 years had more often chronic or mixed malnutrition compared to children between 5 and 10 years. Furthermore, as a marker of HIV-disease progression, children with severe immunodeficiency or recently initiated on ART were more at risk for acute or mixed malnutrition.

Our findings show overall, a high rate of malnutrition in such a healthcare programme. Comparisons with others studies are difficult because of the differences between study population and definitions of malnutrition. Among children aged 2–5 years in our study, 45 % were stunted (chronic + mixed malnutrition) and 14 % were wasted (acute + mixed malnutrition). Compared to a cross-sectional survey conducted in Tanzania among ART-treated HIV-infected children, we observe similar results for wasting but report higher proportions of stunting [19]. The prevalence of malnutrition, whatever the type, was higher than in our study for children initiating ART in Malawi, with more than half of children malnourished [20]. Among children between 2 and 5 years of age, we also report similar rates of malnutrition than those in the general population of children under five years in West and Central Africa (39 % and 12 % for stunting and wasting respectively) [2]. We observed that among HIV-infected children, chronic malnutrition was more frequent than other types. Indeed, because of HIV disease, these children could have underlying problems of nutrition and repeated opportunistic infections.

In our study, children aged 5–10 years suffered less from chronic or mixed malnutrition than children aged 2–5 years. We can hypothesise that the younger children were more advanced in the HIV-disease progression, and were enrolled in the programme precisely because they were more ill, causing malnutrition.

Boys were more likely to be malnourished than girls in our study. A meta-analysis of 10 countries in Sub-Saharan Africa has reported the same result among the general population of children less than five years [21]. In a study conducted in Tanzania among children born to HIV-infected mothers, boys were 28 %, 40 %, and 28 % more likely to be stunted, wasted, and underweight, respectively, compared to girls [22]. The reasons of this association between gender and malnutrition remain unclear and need further investigation [23].

We also noticed that children severely immunodeficient were more likely to be malnourished than non-immunodeficient children, supporting the fact that malnutrition and HIV infection are linked in a vicious circle. Despite this association, we saw that overall half of the malnourished children presented no signs of immunodeficiency. For these children, malnutrition could not be explained by HIV-infection, but rather by an insufficient nutritional support. Moreover, only 30 % of malnourished children were classified at WHO clinical stage III or IV, whereas malnutrition is one of the definitions for stage III and IV for the WHO classification of HIV-related diseases [16], suggesting that malnutrition is not well detected in routine.

We found also an association between recent ART initiation, marker of the HIV-disease progression and mixed malnutrition. We advise caution in interpreting this result since we are unable to access which came first, malnutrition or ART initiation. However, considering previous studies describing the benefits of ART on weight and height gain [24–27]), we hypothesise that these children were probably initiated on ART based on clinical criteria such as malnutrition. Furthermore, we found that non-ART-treated children were more likely to present acute malnutrition compared to ART-treated children.

Similarly, as a marker of malnutrition, children who received a nutritional support six months prior the survey suffered more from chronic malnutrition than those who didn’t received any nutritional support. These children have probably been experiencing malnutrition problems for several months, perhaps several years, leading to chronic malnutrition, which is difficult to reverse.

In our study, orphan status was not associated with malnutrition, which is concordant with other results among HIV-infected children in sub-Saharan Africa [28–30], although other results have reported otherwise [31]. In our context, we can explain our observation by the specific healthcare received by orphaned children in participating associations, reducing differences between orphans and non orphans.

We didn’t find any association between cotrimoxazole prophylaxis and growth in our study, whereas other studies have reported positive effects of such prophylaxis on growth. A study conducted with the Zambian CHAP trial in HIV-infected children, who had not yet received ART, reported that cotrimoxazole prophylaxis slowed decrease in weight and height [32].

This study presents several limitations. First, children included in this study had access to paediatric HIV healthcare, mostly in urban areas where the standard of care may be higher than that offered in rural areas, making results difficult to extrapolate to rural areas. Second, we excluded the children < 2 years, because too few respected the inclusion criteria. However, this population is precisely known to be more vulnerable and having more malnutrition problems [33, 34]. Furthermore, since 50 % of HIV-infected children not initiated on ART die before their second birthday [34], the sickest children could not have survived until the survey period, leading to a survivor bias. So, the selection of the study population is not representative of a birth cohort of HIV-infected children in sub-Saharan Africa, leading to an underestimation of the prevalence of malnutrition. Third, there are possible measurement errors in weight and height; we limited this by using a standard measurement protocol for all centres, following the WHO recommendations [14]. However, peripheral oedema, sign of severe malnutrition, was not collected, despite their effect on increasing artificially weight. Furthermore, because of our definition of malnutrition (acute, chronic and mixed), 20 children with a low weight-for-age were not defined as malnourished and were misclassified in the analyses. Finally, the cross-sectional study design didn’t allow to establish a causal relationship between malnutrition and explanatory variables.

Nevertheless, the study included nearly all children enrolled in the 12 participating associations of the Growing up Programme representing as best as possible HIV-infected children enrolled in HIV care programmes in West and Central Africa. Data collection was of high quality in this study context, with more than 97 % of anthropometric data available. Most of all, very few studies have reported the nutritional practices in HIV-infected malnourished children, and although practices are not detailed in our study, it highlights this gap and the need to focus on these interventions in such children. Finally, despite the possible biases that could have all underestimated the prevalence, we hypothesize that we provided a conservative estimate of the prevalence of malnutrition in a large sample size of Central and West African children, which can give us an idea of the picture of the burden of malnutrition among HIV-infected children in this region.

As a result, we report that anthropometric measurements are not enough routinely performed in the field conditions, and nutritional supplementation is not optimally used and monitored in this context. Indeed, we have seen that close to half of malnourished children didn’t receive any nutritional support, before as well as during the survey, whereas a substantial part of non-malnourished children still had a nutritional support at the moment of the survey. However, taking better into account data on growth in HIV-care programmes could be major to improve long-term paediatric HIV-care.

## Conclusion

In conclusion, the prevalence of malnutrition remains high for HIV-infected children in sub-Saharan Africa, even in an HIV care programme supposed to have a high standard of care. A better acknowledgement of this problem is needed, that should lead to a better healthcare management of HIV-infected children, with active routine anthropometric measurements easy to perform to allow an earlier detection of malnutrition leading to an appropriate nutritional package. Our study strengthens the World Health Organization recommendation on the need for a nutritional assessment and support that should be an integral part of the care plan of HIV-infected children [35]. Indeed, an early detection of growth impairment could detect, for example, poor treatment response, poor adherence to treatment, and could prevent morbidity and mortality risks. Further studies about associated factors with malnutrition, such as differences in sex need to be examined more closely in prospective designs [23]. Moreover, food supplementation and multivitamin use may improve the nutritional status of the children. Finally, nutritional interventions should be tailored and assessed to improve growth, especially at time of ART initiation that could lead to an optimisation of their clinical response and survival of ART-treated children.

## References

[1] UNAIDS (2013). UNAIDS report on the global AIDS.

[2] United Nations Children’s Fund, World Health Organization, and World Bank. UNICEF-WHO-World Bank Joint Child Malnutrition Estimates. New York, USA: UNICEF; Geneva, Switzerland: WHO; and Washington DC, USA: World Bank; 2012

[3] WHO (2012). World Health Statistics 2012.

[4] Victora CG, Adair L, Fall C, Hallal PC, Martorell R, Richter L (2008). Maternal and child undernutrition: consequences for adult health and human capital. Lancet.

[5] Cunningham-Rundles S, McNeeley DF, Moon A (2005). Mechanisms of nutrient modulation of the immune response. J Allergy Clin Immunol.

[6] Romero-Alvira D, Roche E (1998). The keys of oxidative stress in acquired immune deficiency syndrome apoptosis. Med Hypotheses.

[7] Allard JP, Aghdassi E, Chau J, Tam C, Kovacs CM, Salit IE (1998). Effects of vitamin E and C supplementation on oxidative stress and viral load in HIV-infected subjects. AIDS.

[8] Johann-Liang R, O’Neill L, Cervia J, Haller I, Giunta Y, Licholai T (2000). Energy balance, viral burden, insulin-like growth factor-1, interleukin-6 and growth impairment in children infected with human immunodeficiency virus. AIDS.

[9] Trehan I, O’Hare BA, Phiri A, Heikens GT (2012). Challenges in the Management of HIV-Infected Malnourished Children in Sub-Saharan Africa. AIDS Res Treat.

[10] Anema A, Vogenthaler N, Frongillo EA, Kadiyala S, Weiser SD (2009). Food insecurity and HIV/AIDS: current knowledge, gaps, and research priorities. Curr HIV/AIDS Rep.

[11] Saloojee H, De Maayer T, Garenne ML, Kahn K (2007). What’s new? Investigating risk factors for severe childhood malnutrition in a high HIV prevalence South African setting. Scand J Public Health Suppl.

[12] Anabwani G, Navario P (2005). Nutrition and HIV/AIDS in sub-Saharan Africa: an overview. Nutrition.

[13] Chinkhumba J, Tomkins A, Banda T, Mkangama C, Fergusson P (2008). The impact of HIV on mortality during in-patient rehabilitation of severely malnourished children in Malawi. Trans R Soc Trop Med Hyg.

[14] WHO. Training Course on Child Growth Assessment. Module B: Measuring a child’s growth. Geneva, Switzerland: WHO. 2008. Available at: [http://www.who.int/childgrowth/training/module_b_measuring_growth.pdf]

[15] WHO (2010). Antiretroviral therapy for HIV infection in infants and children: Towards universal access. Recommendations for a public health approach, 2010 revision.

[16] WHO (2006). WHO case definitions of HIV for surveillance and revised clinical staging and immunological classification of HIV-related disease in adults and children.

[17] WHO (2006). Multicentre Growth Reference Study Group. WHO Child Growth Standards: length/height-for-age, weight-for-age, weight-for-length, weight-for-height and body mass index-for-age: methods and development.

[18] de Onis M, Onyango AW, Borghi E, Siyam A, Nishida C, Siekmann J (2007). Development of a WHO growth reference for school-aged children and adolescents. Bull World Health Organ.

[19] Sunguya BF, Poudel KC, Otsuka K, Yasuoka J, Mlunde LB, Urassa DP (2011). Undernutrition among HIV-positive children in Dar es Salaam, Tanzania: antiretroviral therapy alone is not enough. BMC Public Health.

[20] Weigel R, Phiri S, Chiputula F, Gumulira J, Brinkhof M, Gsponer T (2010). Growth response to antiretroviral treatment in HIV-infected children: a cohort study from Lilongwe, Malawi. Tropical Med Int Health.

[21] Wamani H, Astrøm AN, Peterson S, Tumwine JK, Tylleskär T (2007). Boys are more stunted than girls in sub-Saharan Africa: a meta-analysis of 16 demographic and health surveys. BMC Pediatr.

[22] McDonald CM, Kupka R, Manji KP, Okuma J, Bosch RJ, Aboud S (2012). Predictors of stunting, wasting and underweight among Tanzanian children born to HIV-infected women. Eur J Clin Nutr.

[23] Muenchhoff M, Goulder PJR (2014). Sex differences in pediatric infectious diseases. J Infect Dis.

[24] Bolton-Moore C, Mubiana-Mbewe M, Cantrell RA, Chintu N, Stringer EM, Chi BH (2007). Clinical outcomes and CD4 cell response in children receiving antiretroviral therapy at primary health care facilities in Zambia. JAMA.

[25] Davies M-A, Keiser O, Technau K, Eley B, Rabie H, van Cutsem G (2009). Outcomes of the South African National Antiretroviral Treatment Programme for children: The IeDEA Southern Africa collaboration. S Afr Med J.

[26] Sutcliffe CG, van Dijk JH, Munsanje B, Hamangaba F, Sinywimaanzi P, Thuma PE (2011). Weight and height z-scores improve after initiating ART among HIV-infected children in rural Zambia: a cohort study. BMC Infect Dis.

[27] McGrath CJ, Chung MH, Richardson BA, Benki-Nugent S, Warui D, John-Stewart GC (2011). Younger age at HAART initiation is associated with more rapid growth reconstitution. AIDS.

[28] Magadi MA (2011). Household and community HIV/AIDS status and child malnutrition in sub-Saharan Africa: evidence from the demographic and health surveys. Soc Sci Med.

[29] Sarker M, Neckermann C, Müller O (2005). Assessing the health status of young AIDS and other orphans in Kampala, Uganda. Trop Med Int Health.

[30] Zidron AM, Juma E, Ice GH (2009). Does being an orphan decrease the nutritional status of Luo children?. Am J Hum Biol.

[31] Watts H, Gregson S, Saito S, Lopman B, Beasley M, Monasch R (2007). Poorer health and nutritional outcomes in orphans and vulnerable young children not explained by greater exposure to extreme poverty in Zimbabwe. Trop Med Int Health.

[32] Prendergast A, Walker AS, Mulenga V, Chintu C, Gibb DM (2011). Improved growth and anemia in HIV-infected African children taking cotrimoxazole prophylaxis. Clin Infect Dis.

[33] Spira R, Lepage P, Msellati P, Van De Perre P, Leroy V, Simonon A (1999). Natural history of human immunodeficiency virus type 1 infection in children: a five-year prospective study in Rwanda. Mother-to-Child HIV-1 Transmission Study Group. Pediatrics.

[34] Newell M-L, Coovadia H, Cortina-Borja M, Rollins N, Gaillard P, Dabis F (2004). Mortality of infected and uninfected infants born to HIV-infected mothers in Africa: a pooled analysis. Lancet.

[35] WHO (2009). Guidelines for an integrated approach to the nutritional care of HIV-infected children (6 months–14 years): handbook, chart booklet and guideline for country adaptation.

